# *Shigella* and Enterotoxigenic *Escherichia coli* Have Replaced Rotavirus as Main Causes of Childhood Diarrhea in Rwanda After 10 Years of Rotavirus Vaccination

**DOI:** 10.1093/infdis/jiae446

**Published:** 2024-09-09

**Authors:** Jean Bosco Munyemana, Jean Claude Kabayiza, Staffan Nilsson, Maria E Andersson, Magnus Lindh

**Affiliations:** Department of Infectious Diseases, Institute of Biomedicine, University of Gothenburg, Sweden; Department of Microbiology and Parasitology, School of Medicine and Pharmacy, University of Rwanda; Department of Pathology, University Teaching Hospital of Kigali; Department of Pediatrics, School of Medicine and Pharmacy, University of Rwanda; Department of Pediatrics, University Teaching Hospital of Kigali, Rwanda; Department of Laboratory Medicine, Institute of Biomedicine, University of Gothenburg, Sweden; Department of Infectious Diseases, Institute of Biomedicine, University of Gothenburg, Sweden; Department of Infectious Diseases, Institute of Biomedicine, University of Gothenburg, Sweden

**Keywords:** children under 5 years, gastroenteritis, genotype, real-time PCR, rotavirus

## Abstract

The causes of diarrhea after 10 years of rotavirus vaccination in Rwanda were investigated with real-time polymerase chain reaction in 496 children with diarrhea and 298 without. Rotavirus was detected in 11% of children with diarrhea (odds ratio, 2.48; *P* = .002). Comparison of population attributable fractions (PAFs) shows that *Shigella* (PAF, 11%) and enterotoxigenic *Escherichia coli* producing labile toxin (PAF, 12%) have replaced rotavirus as the main causative agents. The PAF for rotavirus had declined from 41% prevaccination to 6.5% postvaccination, indicating that rotavirus has become one among several similarly important causes of childhood diarrhea in Rwanda. A rotavirus genotype shift to G3P[8] points at the importance of continued genotype surveillance.

## Teaser

Rotavirus remains a significant cause of diarrhea among children in Rwanda but with a 10-fold lower odds ratio as compared with the period before introduction of rotavirus vaccination and with shifting genotypes.

Acute diarrheal disease remains the second-leading cause of morbidity and mortality in children <5 years of age in developing countries, with global estimates of 1.7 billion cases of diarrhea and >500 000 deaths annually. Rotavirus has been the most important cause of acute diarrhea in children, but the introduction of rotavirus vaccination has substantially reduced the number of cases with severe rotavirus diarrhea and led to a decline in diarrheal disease–associated deaths [1]. Yet, diarrhea continues to be a main reason for pediatric admissions and clinical visits in low- and middle-income countries. Rotavirus appears to remain an important cause of diarrhea in these countries [2], but the reported rotavirus prevalence and the causative role of rotavirus in African countries after the introduction of vaccination vary considerably among studies. Some have reported high rotavirus rates (19%–35%) in sick children but not in controls [3–5], while other have found similar or only slightly reduced rates before and after the introduction of vaccination [6, 7]. A study in Rwanda conducted 2014 to 2015 showed that rotavirus was detected in >30% of vaccinated children with diarrhea but often as a coinfecting pathogen and rarely associated with severe dehydration [8].

The aim of the present study was to investigate the causative role for diarrhea from rotavirus and other pathogens in Rwanda almost 10 years after the introduction of rotavirus vaccination.

## MATERIALS AND METHODS

### Study Design and Area

This cross-sectional study was conducted between 14 September and 5 November 2021 at 9 health facilities representing 4 provinces and Kigali City in Rwanda. The participants were recruited from 9 hospitals, district hospitals, or health care units, as presented in detail in Supplementary Table 1. The aim was to include 500 children with diarrhea and 300 healthy controls.

### Participants and Sample Collection

The patient group included 496 children with diarrhea (at least ≥3 passages of loose or watery stools per day). The control group included 298 children who were free of clinical complaint and without diarrhea at least 96 hours prior to sample collection.

Patients with diarrhea as the chief complaint were recruited during their clinical visits at a health center or hospital, while controls were offered to participate at a scheduled vaccination visit. From each participant, a rectal swab sample (Copan Regular Flocked Swab 502CS01; Copan Italia Spa) was collected in a tube with 1 mL of sterile saline, kept at <8 °C for a few hours, and sent to the University Teaching Hospital of Kigali for storage at −80 °C until being transported to the Department of Infectious Disease, University of Gothenburg, Sweden, where polymerase chain reaction (PCR) analyses were performed.

### Real-time PCR

Nucleic acid purification and 45 cycles of real-time PCR were essentially performed as previously described [8], targeting enterotoxigenic *Escherichia coli* producing labile toxin (ETEC-*eltB*) or stable toxin (ETEC-*estA*), *Shigella*, *Salmonella*, *Campylobacter*, *Cryptosporidium*, rotavirus, adenovirus 40/41, astrovirus, norovirus genogroups I and II, and sapovirus. Additional PCR details, including primers and probes, are presented as supplementary information.

### Rotavirus Genotyping

The 68 samples that were positive for rotavirus were subjected to genotyping by real-time PCR identifying the main G and P subtypes with type-specific primers and probes [8].

### Data Analysis

Statistical analysis was performed with SPSS version 28.0 (IBM Corporation). Fisher exact test was used for comparing prevalence in patients and controls. Multiple logistic regression was used to obtain age-adjusted odds ratios (ORs). Coinfections were investigated by testing if any combination of 2 pathogens was more or less frequent than expected from their detection rates alone. These analyses were performed separately for patients and controls, with statistical significance assessed by Fisher exact test. The population attributable fraction for each pathogen was calculated as follows: prevalence × (1 – 1/OR).

### Ethical Approval

Ethical approvals were obtained from the National Health Research Committee in Rwanda (NHRC/2021/PROT/033) and from the institutional review boards of the Ministry of Health in Rwanda (20/5722/DPMEHF/2021), the University of Rwanda (226/CMHS IRB/2021), and the University Teaching Hospital of Kigali (EC/CHUK/110/2021). A signed consent form was obtained from the child's parents or caretakers after they were informed of the role and nature of the study and before sample collection.

## RESULTS

### Demographics

The children in the patient group were older than the controls (median age, 14.6 vs 9.2 months; *P* < .0001). Females constituted 48.2% of patients and 52.0% of controls. The number of children recruited at the different sites is shown in the Supplementary Table 2.

### Pathogen Detection Rates

**Figure 1.**
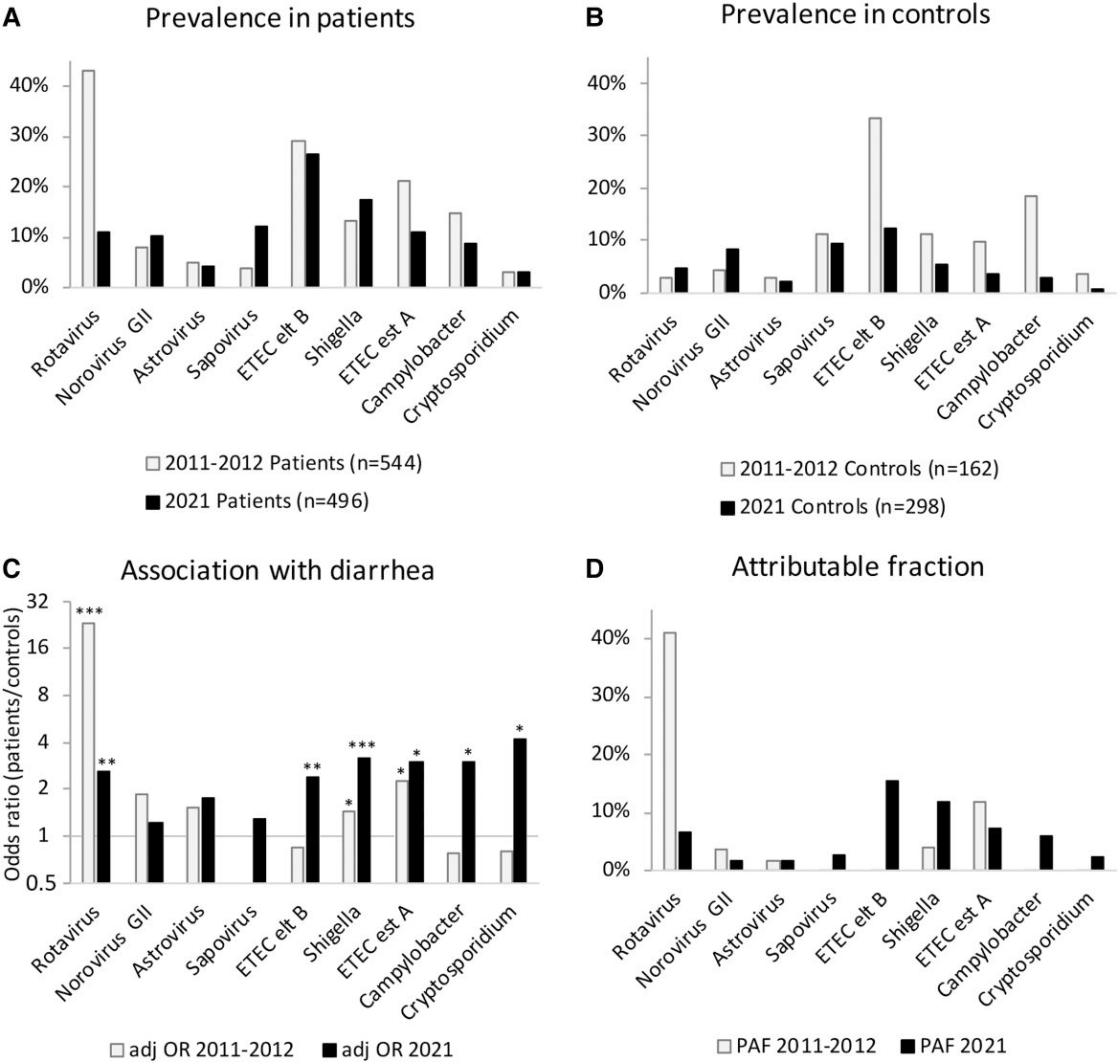
Real-time polymerase chain reaction detection rates of pathogens in children: *A*, patients with diarrhea; *B*, controls. *C*, Increased odds ratios show a causative role for *Shigella*, rotavirus, ETEC-*eltB*, ETEC-*estA*, *Campylobacter*, and *Cryptosporidium*. ***P < .001. **P < .01. *P < .05. *D*, A pronounced reduction for rotavirus and increase for *Shigella* and ETEC-*eltB* of their population attributable fraction, a marker that combines OR and prevalence, thus representing each pathogen's importance as a cause of diarrhea in the population. adj OR, adjusted odds ratio; ETEC-*estA*, *Escherichia coli* producing stable toxin; ETEC-*eltB*, *Escherichia coli* producing labile toxin; PAF, population attributable fraction. The results from the present study (2021) are compared with published results from 2011–2012 [9].

At least 1 pathogen was detected in 336 (68%) patients and 162 (54%) controls. All but 1 of the 803 pathogens that were detected had a cycle threshold value <40. Figure 1 (Supplementary Table 3) shows the detection rates of all pathogens in patients and controls. The ORs indicated a causative role for diarrhea for *Shigella*, rotavirus, ETEC-*eltB*, ETEC-*estA*, *Campylobacter*, and *Cryptosporidium*, and these associations remained significant in multiple logistic regression analysis including all pathogens and age. Comparison with the findings in a similar study in Rwanda from 2011 to 2012 (supplementary information) shows that the prevalence and OR for rotavirus have decreased, whereas ETEC-*eltB* has become especially more important and is more rarely found in controls. Figure 1*D* compares the population attributable fraction of each pathogen, which represents the relative causative importance by combining the OR and prevalence. This comparison identified *Shigella*, ETEC-*eltB*, and ETEC-*estA* as the most important causes of diarrhea. The results indicate that rotavirus remains a significant but no longer predominant causative agent as a causal role. As shown in Table 1, rotavirus was significantly associated with diarrhea in vaccinated and unvaccinated children.

**Table 1.** Detection of Rotavirus in Vaccinated or Unvaccinated Children With or Without Diarrhea

	Diarrhea (Patients; n = 496)^a^	No Diarrhea (Controls; n = 298)^b^	Odds Ratio (95% CI)	*P* Value
Rotavirus PCR+ (n = 68)	54	14	2.48 (1.35–4.54)	.0024
Rotavirus PCR– (n = 726)	442	284		
Vaccinated (n = 722)	440	282	0.41 (.22–.77)	.0034
Not vaccinated (n = 67)	53	14		
Vaccinated (n = 722)				
Rotavirus PCR+ (n = 58)	44	14	2.13 (1.14–3.96)	.017
Rotavirus PCR– (n = 664)	396	268		
Not vaccinated (n = 67)				
Rotavirus PCR+ (n = 8)	8	0	∞ (.46-∞)	.19
Rotavirus PCR– (n = 59)	45	14		

Abbreviation: PCR, polymerase chain reaction.

^a^Vaccination status not known for 3 children with diarrhea.

^b^Vaccination status not known for 2 children without diarrhea.

### Rotavirus Genotypes

G3P[8] (59%) and G9P[8] (16%) were the predominant rotavirus genotypes, whereas low prevalence was observed for G1P[8] (9%), G4P[6] (3%), and G1P[4] (1%). The genotype could not be identified in 8 samples (12%) with low rotavirus concentrations. When compared with the genotypes that we observed in the 2014–2015 study (genotype data provided as Supplementary Table 4), genotype G3P[8] had increased from 0% to 59% (*P* < .0001).

### Coinfections

Coinfections were frequent in patients and controls (Supplementary Table 5). For most pathogen pairs, coinfections were not significantly more frequent than expected from the observed frequency of the pathogens alone. Exceptions were ETEC-*estA* and ETEC-*eltB*, which were positively associated in patients and controls, as anticipated since these toxin genes may be present in the same bacteria. Coinfections with a synergetic impact on symptoms would be associated and have a higher OR in patients but not controls, but that was not observed for any pathogen combination.

## DISCUSSION

In this study of children <5 years of age in Rwanda, at least 1 diarrheal pathogen was detected in 68% of patients and 54% of controls, with ETEC-*eltB* and *Shigella* being the most common. Despite vaccination with 98% reported coverage, rotavirus was detected relatively often (11%) and was significantly associated with diarrhea. However, when compared with a study in Rwanda conducted from 2011 to 2012, before the introduction of rotavirus vaccination [9], the importance of rotavirus as cause of diarrhea was lower, as illustrated by a reduction of the OR from 23.5 to 2.49 and the population attributable fraction from 41.1% to 6.5%. The reduced OR demonstrates that rotavirus infections have become less likely to cause diarrhea, and the lower prevalence among patients (11% vs 43% in 2011–2012) indicates that vaccination for a decade may have also reduced the circulation of rotavirus. In a study conducted in 2014, two years after implementation of vaccination, the rate of rotavirus among children with diarrhea was still as high as 34% [8]. Seven more years of vaccination may have reduced the circulation of rotavirus in Rwanda, but it is likely that improvements in hygiene, sanitation, and water supply, in combination with COVID-19 restrictions, also have contributed to the lower frequency of rotavirus infection in the present study.

Although it is well established that rotavirus vaccination reduces severity of symptoms [10], the effect of vaccination in African countries needs further study [11]. Some previous studies suggest that rotavirus remains an important cause of diarrhea in vaccinated populations, but the data are conflicting. Studies from Malawi and Tanzania identified rotavirus in 35% of children with diarrhea and in only 1.5% of controls (OR, 23) [3], and a study from Mozambique [6] found a similar rotavirus frequency before and after the introduction of vaccination and no protective effect on symptoms (OR, 1.93 before vaccine introduction; OR, 2.27 after) [6]. Both these studies indicate a poor effect of vaccination in these countries. An incomplete protection might be due to incomplete vaccination coverage, a poorer vaccine response in malnourished children, or a mismatch between the vaccine and circulating rotavirus genotypes [8, 12].

In the present study, rotavirus was significantly associated with diarrhea in vaccinated children, but the markedly reduced population attributable fraction demonstrates a profound effect by vaccination on rotavirus morbidity in Rwanda where the officially reported vaccination coverage is 98%. However, the protective effect of the vaccine might decline in the future if new genotypes emerge, a possibility suggested by observed genotype shifts. Lower frequencies of G1P[8] and G2P[4] have been reported by others after the introduction of vaccination [12], and from 2014 to 2015, we observed a shift from predominance of G9P[8] from 2011 to 2012 to G12[P8]. In the present study, we instead found predominance of G3P[8] and reemergence of G9P[8].

Our findings of increased population attributable fraction values for ETEC*-eltB*, *Shigella*, and ETEC*-estA* point at an increase of these pathogens as causes of diarrhea [13]. ETEC*-eltB* was detected in 27% and 12% (*P* = .0001) of children with or without diarrhea, as compared with 29% and 33% from 2011 to 2012. Previous studies have found that ETEC*-eltB* may cause diarrhea in low-income countries, especially during the first year of life, whereas in older children this infection is often asymptomatic [9, 14], probably as a result of accumulating immunity. A delayed primary ETEC*-eltB* infection due to improved hygiene in combination with COVID-19 restrictions might explain its strong association with diarrhea in the present study, in which 80% of the children were older than 1 year. Improved hygiene and COVID-19 restriction might likewise explain that ETEC*-estA* and *Campylobacter* infections were half as common as in the earlier study. This observation argues that efforts to improve hygiene and general living conditions might be the most effective way to reduce acute diarrhea in low- and middle-income countries, but vaccine development against ETEC, *Campylobacter*, and *Shigella* is also of high priority. *Shigella* was detected in 17% and 5% in children with and without diarrhea (*P* = .0001), as compared with 13% and 11% from 2011 to 2012. In the present study, the patients were significantly older than controls, but the strong association between *Shigella* and diarrhea remained when age was considered.

A limitation in the present study is the relatively short sampling period (September–October), whereas the former study (2011–2012) collected samples during several months. However, the rotavirus positivity rate was lower if only September–October data were considered (51% in 2011, 11% in 2021). Moreover, the strong decline in OR for rotavirus should be independent of differences in the sampling collection date.

In summary, we found a generally lower frequency of diarrheagenic pathogens than in earlier studies in Rwanda and propose that this can be a result of COVID-19 restrictions and socioeconomic development with improved hygiene, sanitation, and water supply. An effect of rotavirus vaccination was observed as a lower prevalence of rotavirus and a relative increase of rotavirus infections without diarrhea, resulting in a strong reduction of the population attributable fraction for rotavirus. Despite that, rotavirus remains a significant cause of diarrhea in Rwanda, and continued surveillance of genotypes is important because future genotype shifts might reduce the protective effect of the vaccine. The observed increased importance of ETEC and *Shigella* emphasize the need to reduce these infections [13] by vaccine development [15], safe water, and improved hygiene and sanitation.

## Supplementary Data

Supplementary materials are available at *The Journal of Infectious Diseases* online (http://jid.oxfordjournals.org/). Supplementary materials consist of data provided by the author that are published to benefit the reader. The posted materials are not copyedited. The contents of all supplementary data are the sole responsibility of the authors. Questions or messages regarding errors should be addressed to the author.

## Supplementary Material

jiae446_Supplementary_Data

## Notes

***Acknowledgments.*** We thank the nurses and laboratory personnel in Rwanda for registering data and collecting samples from the children.

***Author contributions.*** J. B. M. conducted the field studies, performed PCR analysis, analyzed data, and drafted the manuscript. J. C. K. planned and supervised the field studies. S. N. performed statistical analysis. M. E. A. planned the study, performed PCR, and genotyping analysis. M. L. planned the study, analyzed the data, and finalized the manuscript. All authors reviewed the final manuscript.

***Financial support.*** This work was supported by grants from the Swedish International Development Cooperation Agency (SIDA 2019/37:9) to M. L.
