# Supplementary material for: Shigella and Enterotoxigenic Escherichia coli Have Replaced Rotavirus as Main Causes of Childhood Diarrhea in Rwanda After 10 Years of Rotavirus Vaccination
Source: J Infect Dis. 2024 Sep 9;230(5):e1176–80. doi: 10.1093/infdis/jiae446 (PMC11566240; doi:10.1093/infdis/jiae446)
Supplement: jiae446_Supplementary_Data [file jiae446_supplementary_data.zip › Supplementary_Table1_primers&probes.docx]

**Supplementary Table 1. Primers and probes used for real-time PCR in eight parallel reaction**

|  | Mix | Forward primer | Reverse primer | Probe | |
| --- | --- | --- | --- | --- | --- |
| Norovirus GII | 1 | TGGAYTTTTAYGTGCCCAG | CGACGCCATCTTCATTCAC | VIC-AGCCAGATTGCGATCRCCC-TAMRA | |
| Rotavirus | 1 | AACCATCTACACATGACCCTCTATGA | GGTCACATAACGCCCCTATAGC | FAM-CAATAGTTAAAAGCTAACACTGTCAAA-MGB | |
|  | 1 | AACCATCTTCACGTAACCCTCTATGA |  |  | |
| Astrovirus | 2 | GACTGCWAAGCAGCTTCGTGA | GCTAGCCATCACACTTCTTTGGTCCT | FAM-TCACAGAAGAGCAACTCCATCGCATTTG-BQ1 | |
| Sapovirus | 2 | TTGGCCCTCGCCACCTAC | CCCTCCATYTCAAACACTA | VIC-CCRCCTATRAACCA-MGB | |
|  | 2 | GAYCASGCTCTCGCYACCTAC |  |  | |
| Norovirus GI | 3 | TGGCAGGCCATGTTCCGCT | TTTGKTGGGGCGTCCTTAGAC | VIC-ATTGCGATCTCCTGTCCA-MGB | |
|  | 3 |  | CGCTTGATGTAGCGTCCTTAGAC |  | |
|  | 3 |  | AGGCTCAGCTGTATTTGCCTCTGGT |  | |
| *Campylobacter jejuni* | 4 | ATGCAAACCATAATTGGGTTTCAAC | CGAGTATCAGCAACTTCTTCTACAGCT | NED-TTGCCACCAAAACCAAAACT-MGB | |
| *Salmonella* | 5 | CGGGTTGCGTTATAGGTCTGA | TGAAATACGATGCGAACAACATC | VIC-AATACTGCGCTGCCAGAT-MGB | |
| ETEC*-estA* | 5 | AAGCATGAATAGTAGCAATTACTGCT | TTAATAGCACCCGGTACAAGCA | NED-AACAACACAATTCAC-MGB | |
| ETEC*-eltB* | 6 | TCCGGCAGAGGATGGTTACA | CCAGGGTTCTTCTCTCCAAGC | FAM-AGCAGGTTTCCCACCGGATCACC-BQ1 | |
| *Shigella* | 6 | ACCGGCGCTCTGCTCTC | GCAATGTCCTCCAGAATTTCG | JOE-CTGGGCAGGGAAATGTTCCGCC-BQ1 | |
| *Cryptosporidium parvum/hominis* | 7 | CAAATTGATACCGTTTGTCCTTCTG | TGGTGCCATACATTGTTGTCCT | NED-TGTCCTCCTGGATTCA-MGB | |
| Adenovirus 40/41 | 8 | TGCCCGCGCCACCGAT | GAGCCACAGTGGGGTTTCTG | NED-CCAGGCTGAAGTACG-BQ1 |  |

MGB, minor groove binding. All probe concentrations were 200 nM. Primer concentrations were 900 nM for astrovirus and norovirus GI, 300 nM for all other agents.
