# Supplementary material for: Shigella and Enterotoxigenic Escherichia coli Have Replaced Rotavirus as Main Causes of Childhood Diarrhea in Rwanda After 10 Years of Rotavirus Vaccination
Source: J Infect Dis. 2024 Sep 9;230(5):e1176–80. doi: 10.1093/infdis/jiae446 (PMC11566240; doi:10.1093/infdis/jiae446)
Supplement: jiae446_Supplementary_Data [file jiae446_supplementary_data.zip › Supplementary_Table2_studysites_age_sex.docx]

**Supplementary Table 2. Study sites and demographics of the participants**

|  |  | Patients (n=496) | Controls (n=298) | Total  (n=794) |
| --- | --- | --- | --- | --- |
| Study Sites | CHUK | 60 | 51 | 111 |
|  | Gisenyi DH | 56 | 0 | 56 |
|  | Kabutare DH | 104 | 0 | 104 |
|  | Kacyiru Hospital | 26 | 37 | 63 |
|  | Kagugu HC | 5 | 93 | 98 |
|  | Legacy Clinic | 30 | 0 | 30 |
|  | Nyacyonga HC | 37 | 63 | 100 |
|  | Ruhengeri RH | 93 | 7 | 100 |
|  | Rwamagana PH | 85 | 47 | 132 |
|  |  |  |  |  |
| Sex | Female | 239 | 155 | 394 |
|  | Male | 257 | 143 | 400 |
|  |  |  |  |  |
| Age | Mean age (months) | 18.8 | 14.8 | 17.3 |
|  | Median age (months) | 14.6 | 9.2 | 13.0 |
|  |  |  |  |  |
| Age groups | Under 6 months | 119 | 122 | 241 |
|  | 6–11 months | 108 | 49 | 157 |
|  | 12–23 months | 146 | 62 | 208 |
|  | 24–35 months | 62 | 32 | 94 |
|  | 36–47 months | 32 | 21 | 53 |
|  | 48–59 months | 29 | 12 | 41 |
|  |  |  |  |  |
| HIV status | Negative | 335 | 219 | 554 |
|  | Positive | 9 | 1 | 10 |
|  | Not known | 150 | 76 | 226 |

CHUK, University Teaching Hospital of Kigali; DH, District Hospital; HC; Health Centre; RH, Referral Hospital; PH, Provincial Hospital; HIV, Human Immunodeficiency Virus.
