# Supplementary material for: Shigella and Enterotoxigenic Escherichia coli Have Replaced Rotavirus as Main Causes of Childhood Diarrhea in Rwanda After 10 Years of Rotavirus Vaccination
Source: J Infect Dis. 2024 Sep 9;230(5):e1176–80. doi: 10.1093/infdis/jiae446 (PMC11566240; doi:10.1093/infdis/jiae446)
Supplement: jiae446_Supplementary_Data [file jiae446_supplementary_data.zip › Supplementary_Table3_detection_rates.docx]

**Supplementary Table 3. Detection frequencies in patients and controls**

|  | Patients (n=496) | | Controls (n=298) | | OR | P value | Multiple logistic regression P value^a^ |
| --- | --- | --- | --- | --- | --- | --- | --- |
| *Shigella* | 86 | 17.3% | 16 | 5.4% | 3.7 | <0.0001 | 0.0001 |
| Rotavirus | 54 | 10.9% | 14 | 4.7% | 2.48 | 0.0024 | 0.0023 |
| ETEC-*eltB* | 132 | 26.6% | 37 | 12.4% | 2.56 | <0.0001 | 0.0032 |
| ETEC-*estA* | 54 | 10.9% | 11 | 3.7% | 3.19 | 0.0003 | 0.038 |
| *Campylobacter* | 43 | 8.7% | 9 | 3.0% | 3.05 | 0.0016 | 0.011 |
| *Cryptosporidium* | 15 | 3,0% | 2 | 0.7% | 4.62 | 0.039 | 0.045 |
| Astrovirus | 21 | 4.2% | 7 | 2.3% | 1.84 | 0.2 | NS |
| Norovirus GI | 21 | 4.2% | 9 | 3.0% | 1.42 | 0.44 | NS |
| Sapovirus | 61 | 12.3% | 28 | 9.4% | 1.35 | 0.25 | NS |
| Norovirus GII | 50 | 10.1% | 25 | 8.4% | 1.22 | 0.46 | NS |
| *Salmonella* | 33 | 6.7% | 19 | 6.4% | 1.05 | 1 | NS |
| Adenovirus 40/41 | 35 | 7.1% | 21 | 7.0% | 1 | 1 | NS |

^a^ With presence of diarrhea as dependent variable and *Shigella*, rotavirus, ETEC-*eltB*, ETEC-*estA*, *Campylobacter*, *Cryptosporidium,* and age as independent variables.
