# Supplementary material for: Shigella and Enterotoxigenic Escherichia coli Have Replaced Rotavirus as Main Causes of Childhood Diarrhea in Rwanda After 10 Years of Rotavirus Vaccination
Source: J Infect Dis. 2024 Sep 9;230(5):e1176–80. doi: 10.1093/infdis/jiae446 (PMC11566240; doi:10.1093/infdis/jiae446)
Supplement: jiae446_Supplementary_Data [file jiae446_supplementary_data.zip › Supplementary_Table4_Rotagenotypes2014-2015.docx]

Supplementary Table 4. Rotavirus genotypes observed 2014-2015 in Rwandan children <5 years with diarrhea (Kabayiza et al. Rotavirus infections and their genotype distribution in Rwanda before and after the introduction of rotavirus vaccination. PLoS One 2023; 18:e0284934).

|  | G1P8 | G2P4 | G4P8 | G8P4 | G9P8 | G12P6 | G12P8 | Other |
| --- | --- | --- | --- | --- | --- | --- | --- | --- |
| 2014, unvaccinated (n=170) | 6% | 0% | 11% | 11% | 6% | 0% | 61% | 6% |
| 2014, vaccinated (n=18) | 0% | 0% | 15% | 2% | 0% | 1% | 68% | 14% |
| 2015, vaccinated (n=64) | 30% | 0% | 2% | 0% | 6% | 0% | 48% | 14% |
