# Supplementary material for: Shigella and Enterotoxigenic Escherichia coli Have Replaced Rotavirus as Main Causes of Childhood Diarrhea in Rwanda After 10 Years of Rotavirus Vaccination
Source: J Infect Dis. 2024 Sep 9;230(5):e1176–80. doi: 10.1093/infdis/jiae446 (PMC11566240; doi:10.1093/infdis/jiae446)
Supplement: jiae446_Supplementary_Data [file jiae446_supplementary_data.zip › Supplementary_Table5_coinfections.docx]

**Supplementary Table 5**. Co-infections among children without (controls) or with (patients) diarrhea. For each pathogen combination, the number of cases with this co-infection is presented first, followed by the odds ratio and the p value.

|  |  |  | Adenovirus | Astrovirus | NoV GI | NoV GII | Rotavirus | *Sapovirus* | *Cryptosporidium* | *Campylobacter* | ETEC*-eltB* | ETEC*-estA* | *Salmonella* |
| --- | --- | --- | --- | --- | --- | --- | --- | --- | --- | --- | --- | --- | --- |
|  |  |  | number of co-detected/OR/p value | | | | | | | | | | |
| **Healthy controls (n=298)** | |  | 21 | 7 | 9 | 25 | 14 | 28 | 2 | 9 | 37 | 11 | 19 |
| Adenovirus | 21 | number co-detected; OR; p value |  |  |  |  |  |  |  |  |  |  |  |
| Astrovirus | 7 |  | 1; 2.26; 0.41 |  |  |  |  |  |  |  |  |  |  |
| NoV GI | 9 |  | 2; 4.06; 0.126 | 0; 0; 1 |  |  |  |  |  |  |  |  |  |
| NoV GII | 25 |  | 2; 1.62; 0.69 | 0; 0; 1 | 0; 0; 1 |  |  |  |  |  |  |  |  |
| Rotavirus | 14 |  | 2; 2.32; 0.26 | 0; 0; 1 | 1; 2.65; 0.36 | 1; 0.83; 1 |  |  |  |  |  |  |  |
| Sapovirus | 28 |  | 4; 2.48; 0.12 | 2; 4.08; 0.13 | 1; 1.21; 0.59 | 2; 0.82; 1 | 4; 4.33; 0.032 |  |  |  |  |  |  |
| *Cryptosporidium* | 2 |  | 0; 0; 1 | 0; 0; 1 | 0; 0; 1 | 1; 11.33; 0.16 | 0; 0; 1 | 0; 0; 1 |  |  |  |  |  |
| *Campylobacter* | 9 |  | 2; 4.06; 0.13 | 0; 0; 1 | 0; 0; 1 | 1; 1.38; 0.55 | 0; 0; 1 | 1; 1.21; 0.59 | 1; 36; 0.059 |  |  |  |  |
| ETEC*-eltB* | 37 |  | 6; 3.17; 0.032 | 2; 2.92; 0.21 | 4; 6.2; 0.016 | 3; 0.96; 1 | 3; 2.0; 0.39 | 5; 1.62; 0.37 | 0; 0; 1 | 0; 0; 0.61 |  |  |  |
| ETEC*-estA* | 11 |  | 2; 3.13; 0.18 | 1; 4.68; 0.23 | 1; 3.49; 0.29 | 1; 1.09; 1 | 1; 2.11; 0.42 | 2; 2.23; 0.28 | 0; 0; 1 | 0; 0; 1 | **7; 15; <0,0001** |  |  |
| *Salmonella* | 19 |  | 4; 4.11; 0.035 | 0; 0; 1 | 0; 0; 1 | 1; 0.59; 1 | 1; 1.14; 1 | 3; 1.91; 0.40 | 0; 0; 1 | 0; 0; 1 | 5; 2.76; 0.07 | 0; 0; 1 |  |
| *Shigella* | 16 |  | 0; 0; 0.61 | 1; 3.07; 0.32 | 1; 2.28; 0.40 | 2; 1.61; 0.63 | 0; 0; 1 | 1; 0.63; 1 | 0; 0; 1 | 0; 0; 1 | 5; 3.55; 0.035 | 0; 0; 1 | 0; 0; 0.61 |
|  |  |  |  |  |  |  |  |  |  |  |  |  |  |
| **Patients (n=496)** |  |  | 35 | 21 | 21 | 50 | 54 | 61 | 15 | 43 | 132 | 54 | 33 |
| Adenovirus | 35 | number co-detected;OR;p value |  |  |  |  |  |  |  |  |  |  |  |
| Astrovirus | 21 |  | 3; 2.31; 0.18 |  |  |  |  |  |  |  |  |  |  |
| NoV GI | 21 |  | 0; 0; 0.37 | 0; 0; 1 |  |  |  |  |  |  |  |  |  |
| NoV GII | 50 |  | 3; 0.83; 1 | 1; 0.43; 0.71 | 1; 0.43; 0.71 |  |  |  |  |  |  |  |  |
| Rotavirus | 54 |  | 3; 0.75; 1 | 4; 2; 0.27 | 0; 0; 0.15 | 2; 0.32; 0.15 |  |  |  |  |  |  |  |
| Sapovirus | 61 |  | 3; 0.65; 0.60 | 3; 1.20; 0.73 | 3; 1.20; 0.73 | 4; 0.59; 0.49 | 2; 0.25; 0.046 |  |  |  |  |  |  |
| *Cryptosporidium* | 15 |  | 1; 0,94; 1 | 0; 0; 1 | 0; 0; 1 | 0; 0; 0.38 | 0; 0; 0.39 | 2; 1.10; 0.71 |  |  |  |  |  |
| *Campylobacter* | 43 |  | 3; 0.98; 1 | 4; 2.63; 0.099 | 3; 1.81; 0.41 | 5; 1.19; 0.79 | 9; 2.4; 0.038 | 4; 0.71; 0.80 | 3; 2.75; 0.13 |  |  |  |  |
| ETEC-*eltB* | 132 |  | 13; 1.70; 0.16 | 4; 0.64; 0.61 | 8; 1.74; 0.21 | 18; 1.64; 0.13 | 14; 0.96; 1 | 20; 1.41; 0.28 | 4; 1.0; 1 | 15; 1.54; 0.21 |  |  |  |
| ETEC-*estA* | 54 |  | 6; 1.78; 0.25 | 6; 3.56; 0.019 | **10; 8.90; <0.0001** | 3; 0.49; 0.33 | 8; 1.49; 0.35 | 8; 1.28; 0.51 | 3; 2.11; 0.22 | 3; 0.59; 0.61 | **35; 6.55; <0.0001** |  |  |
| *Salmonella* | 33 |  | 1; 0.39; 0.50 | 0; 0; 0.39 | 0; 0; 0.39 | 6; 2.12; 0.13 | 0; 0; 0.038 | 1; 0.21; 0.10 | 0; 0; 1 | 3; 1.06; 1 | **16; 2.81; 0.0068** | 3; 0.80; 1 |  |
| *Shigella* | 86 |  | 7; 1.21; 0.65 | 6; 1.98; 0.23 | 6; 1.96; 0.23 | 7; 0.75; 0.69 | 5; 0.45; 0.13 | 10; 0.91; 1 | 3; 1.2; 0.73 | 10; 1.50; 0.29 | **36; 2.36; 0.0007** | 14; 1.80; 0.087 | 10; 2.21, 0.054 |
