# Supplementary material for: Shigella and Enterotoxigenic Escherichia coli Have Replaced Rotavirus as Main Causes of Childhood Diarrhea in Rwanda After 10 Years of Rotavirus Vaccination
Source: J Infect Dis. 2024 Sep 9;230(5):e1176–80. doi: 10.1093/infdis/jiae446 (PMC11566240; doi:10.1093/infdis/jiae446)
Supplement: jiae446_Supplementary_Data [file jiae446_supplementary_data.zip › Suppl_info_PCR.docx]

**Real-time PCR testing of rectal swab samples**

**Sample processing and nucleic acid purification**

External lysis was performed by mixing approximately 250 μL of the rectal swab with 2 mL of lysis buffer, and this volume was used for extraction of total nucleic acid in an EasyMag instrument (Biomerieux, Marcy l’Étoile, France). The nucleic acids were eluted in 110 μL volume, and 5 μL of this were used for each PCR.

**Amplification**

Amplification was performed in a Quantstudio 6 instrument (Applied Biosystems, Foster City, CA) in 8 parallel 20-μL reactions that contained oligonucleotide described in Supplementary Table 2.

The Taqman Fast Virus 1-step Mastermix (ABI, for RNA targets) and Universal Mastermix (ABI, for DNA targets) were mixed with primers and probes in a final concentration of 300 nM (900 nM for astrovirus and norovirus GI) and 200 nM, respectively. Amplification (15 seconds at 95°C, 60 seconds at 56°C) was run for 45 cycles after an initial 30-minute reverse transcription step at 46°C and 10-minute denaturation at 95°C. In each run, plasmids containing the target regions for all agents were amplified in parallel with study specimens to verify the performance of each target PCR.
