# Supplementary material for: Shigella and Enterotoxigenic Escherichia coli Have Replaced Rotavirus as Main Causes of Childhood Diarrhea in Rwanda After 10 Years of Rotavirus Vaccination
Source: J Infect Dis. 2024 Sep 9;230(5):e1176–80. doi: 10.1093/infdis/jiae446 (PMC11566240; doi:10.1093/infdis/jiae446)
Supplement: jiae446_Supplementary_Data [file jiae446_supplementary_data.zip › Suppl_info_study2011-2012.docx]

Supplementary information about a previous study conducted 2011-2012 in Rwanda (Kabayiza et al,. Real-time PCR identification of agents causing diarrhea in Rwandan children less than 5 years of age. Pediatr Infect Dis J 2014; 33:1037-42).

**Patients**

The patients (n=544) were included among children seeking care in 2 health centers, 2 district hospitals and 2 university hospitals.

Inclusion criteria: age ≤5.0 years and diarrhea with a duration of <96 hours (with or without vomiting or fever).

Diarrhea was defined as 3 or more loose or watery stools per day, but in breastfeeding infants, diarrhea was considered when they had more than 6 stools per day.

**Healthy Controls**

The controls (n=162) were recruited by nurses and community health workers at nursing schools and immunization centers. They were healthy children below 5.0 years of age, living in the same geographic area as the patients, without any episode of diarrhea in 2 weeks prior to the sampling date.

The participants (patients and controls) were included during both the main raining season (from March to May) and the main dry season (July to August) in 2011–2012.

**PCR procedure**

For faeces samples, the faeces (approximately 250 μL) was dissolved in 4.5 mL of saline and centrifuged 5 minutes at 750x g. Then, 250 μL of the dissolved faeces or 250 μL of the rectal swab solution were mixed with 2 mL of lysis buffer, and this volume was used for extraction of total nucleic acid in an EasyMag instrument (Biomerieux, Marcy l’E´toile, France). The nucleic acids were eluted in 110 μ L of which 5 μL were used for each multiplex real-time PCR.

Real-time PCR was performed in an ABI 7900 384-well system (Applied Biosystems, Foster City, CA) in 20 μL-reactions containing oligonucleotides and TaqMan Fast Virus 1-step Master mix (ABI, for RNA targets) or Universal Master mix (ABI, for DNA targets). After a reverse transcription step at 46˚C for 30 min followed by 10 min of denaturation at 95˚C, 45 cycles of two-step PCR was performed (15 s at 95˚C, 60 s at 56˚C). In each run, plasmids containing the target regions for all agents were amplified in parallel with patient specimens

to verify the performance of each target PCR.

**Primers and probes**

|  | Mix | Forward primer | Reverse primer | Probe | Fluoro-phore | Target (gene/region) |
| --- | --- | --- | --- | --- | --- | --- |
| Norovirus GG2 | 1 | TGGAYTTTTAYGTGCCCAG | CGACGCCATCTTCATTCAC | AGCCAGATTGCGATCGCCC | VIC-TAMRA | pol-capsid junction |
| Rotavirus | 1 | AACCATCTACACATGACCCTCTATGA | GGTCACATAACGCCCCTATAGC | CAATAGTTAAAAGCTAACACTGTCAAA | FAM-MGB | NSP3 |
|  | 1 | AACCATCTTCACGTAACCCTCTATGA |  |  |  |  |
| Astrovirus | 2 | GACTGCWAAGCAGCTTCGTGA | GCTAGCCATCACACTTCTTTGGTCCT | TCACAGAAGAGCAACTCCATCGCATTTG | FAM-BQ1 | pol-capsid junction |
| Sapovirus | 2 | TTGGCCCTCGCCACCTAC | CCCTCCATYTCAAACACTA | CCRCCTATRAACCA | VIC-MGB | pol-capsid junction |
|  | 2 | GAYCASGCTCTCGCYACCTAC |  |  |  |  |
| Norovirus GG1 | 3 | TGGCAGGCCATGTTCCGCT | TTTGKTGGGGCGTCCTTAGAC | ATTGCGATCTCCTGTCCA | VIC-MGB | pol-capsid junction |
|  | 3 |  | CGCTTGATGTAGCGTCCTTAGAC |  |  |  |
| Campylobacter jejuni | 4 | ATGCAAACCATAATTGGGTTTCAAC | CGAGTATCAGCAACTTCTTCTACAGCT | TTGCCACCAAAACCAAAACT | NED-MGB | cadF |
| Yersinia enterocolitica | 5 | GCTKGATTGTCAGGAGTTGGTC | ATCCCCCGCAGTTGGCAT | ACCCGCTAATGAAGCA | VIC-MGB | Yst precursor |
| Vibrio cholerae | 6 | CCACTTAGTGGGTCAAACTATATTGTC | ATGCCCCTAATACATCATTAACGTT | AGCCACTGCACCCAA | FAM-MGB | ctxA |
| Salmonella spp | 7 | CGGGTTGCGTTATAGGTCTGA | TGAAATACGATGCGAACAACATC | AATACTGCGCTGCCAGAT | VIC-MGB | ompC |
| ETEC estA | 7 | AAGCATGAATAGTAGCAATTACTGCT | TTAATAGCACCCGGTACAAGCA | AACAACACAATTCAC | NED-MGB | estA |
| ETEC eltB | 8 | TCCGGCAGAGGATGGTTACA | CCAGGGTTCTTCTCTCCAAGC | AGCAGGTTTCCCACCGGATCACC | FAM-BQ1 | eltB |
| Shigella spp | 8 | ACCGGCGCTCTGCTCTC | GCAATGTCCTCCAGAATTTCG | CTGGGCAGGGAAATGTTCCGCC | JOE-BQ1 | ipaH |
| Cryptosporidium parvum/hominis | 9 | CAAATTGATACCGTTTGTCCTTCTG | TGGTGCCATACATTGTTGTCCT | TGTCCTCCTGGATTCA | NED-MGB | OWP |
| E coli eae | 9 | ACATGACCGATGACAAGGCA | CGCGACTGAAGCTGGCTAC | TCGCCGCCTGTTGTGCCG |  | eae |
| E coli bfpa | 9 | GGTCTGTCTTTGATTGAATCTGCA | GCAGACTGGTAGTAAAACATCACACC | GCGCTTGCTGCCACCGTTACCG |  | bpfA |
| Adenovirus | 10 | GCCACGGTGGGGTTTCTAAACTT | GCCCCAGTGGTCTTACATGCACATC | TGCACCAGACCCGGGCTCAGGTACTCCGA | FAM-BQ1 | hexon |

MGB, minor groove binding.

**Detection rates by real-time PCR**

|  | Patients (n=544) | | Controls (n=162) | | OR | P value^a^ |  |
| --- | --- | --- | --- | --- | --- | --- | --- |
|  | n | % | n | % |  |  |  |
| Adenovirus | 216 | 40% | 68 | 42% | 0.91 | 0.64 |  |
| Astrovirus | 26 | 4.8% | 5 | 3.1% | 1.58 | 0.51 |  |
| Norovirus GG1 | 15 | 2.8% | 12 | 7.4% | 0.35 | 0.02 |  |
| Norovirus GG2 | 44 | 8.1% | 7 | 4.3% | 1.95 | 0.12 |  |
| Rotavirus | 233 | 43% | 5 | 3.1% | 23.52 | <0.0001 |  |
| Sapovirus | 20 | 3.7% | 18 | 11% | 0.31 | 0.0006 |  |
| Cryptosporidium | 17 | 3.1% | 6 | 3.7% | 0.84 | 0.80 |  |
| Campylobacter | 81 | 15% | 30 | 19% | 0.77 | 0.27 |  |
| ETEC eltB | 159 | 29% | 54 | 33% | 0.83 | 0.33 |  |
| ETEC estA | 114 | 21% | 16 | 10% | 2.42 | 0.001 |  |
| EPEC bfpa | 53 | 10% | 13 | 8.0% | 1.24 | 0.64 |  |
| EPEC eae | 120 | 22% | 47 | 29% | 0.69 | 0.07 |  |
| Salmonella | 29 | 5.3% | 17 | 10% | 0.48 | 0.03 |  |
| Shigella ipaH | 72 | 13% | 18 | 11% | 1.22 | 0.59 |  |

^a^ Fisher’s exact test
